# The Effect of Continuous Light on Growth and Muscle-Specific Gene Expression in Atlantic Salmon (*Salmo salar* L.) Yearlings

**DOI:** 10.3390/life11040328

**Published:** 2021-04-10

**Authors:** Natalia S. Shulgina, Maria V. Churova, Svetlana A. Murzina, Marina Yu. Krupnova, Nina N. Nemova

**Affiliations:** Environmental Biochemistry Lab, Institute of Biology of the Karelian Research Centre of the Russian Academy of Sciences, Pushkinskaya st., 11, 185910 Petrozavodsk, Russia; murzina.svetlana@gmail.com (S.A.M.); mukrupnova@rambler.ru (M.Y.K.); nnnemova@gmail.com (N.N.N.)

**Keywords:** Atlantic salmon, muscle-specific gene expression, growth, photoperiod regimes

## Abstract

Photoperiod is associated to phenotypic plasticity of somatic growth in several teleost species, however, the molecular mechanisms underlying this phenomenon are currently unknown. The effect of a continuous lighting (LD 24:0), compared with the usual hatchery lighting (HL) regime, on the growth rate and gene expression of myogenic regulatory factors (MRFs: *MyoD1* paralogs, *Myf5*, and *MyoG*) myosin heavy chain (*MyHC*), and *MSTN* paralogs in the white muscles of hatchery-reared Atlantic salmon yearlings was evaluated over a 6-month period (May–October). The levels of gene expression were determined using real-time PCR. Continuous lighting was shown to have a positive effect on weight gain. *MyHC*, *MyoD1c*, *MyoD1b*, and *MSTN1a/b* mRNA expression was influenced by the light regime applied. In all the studied groups, a significant positive correlation was observed between the expression levels of MRFs and *MSTN* paralogs throughout the experiment. The study demonstrated seasonal patterns regarding the simultaneous expression of several MRFs. *MyoD1a*, *MyoG*, and *MyHC* mRNA expression levels were elevated in the mid-October, but *MyoD1b/c*, and *Myf5* mRNA levels decreased by the end of this month. In general, the findings showed that constant lighting affected the regulatory mechanisms of muscle growth processes in salmon.

## 1. Introduction

Postembryonic muscle growth in fish, unlike that in birds and mammals, is determined not only by hypertrophy, an increase in the size of existing muscle fibers, but also by hyperplasia, an increase in the number of muscle fibers due to the recruitment of new myotubes [1]. Both these processes continue throughout the fish lifecycle and are controlled by the sequential expression of myogenic regulatory factors (MRFs), transcription factors of the bHLH family. The MRFs family includes genes transcripts—*MyoD*, *Myf5*, myogenin, and *MRF4* [2]. In the initial stages of muscle growth, myoblast proliferation and cell hyperplasia are activated by the high expression of *MyoD* and *Myf5*, while the expression of myogenin and *MRF4* is most pronounced in adults and is associated with myoblast differentiation and muscle fiber hypertrophy (i.e., an increase in the number of nuclei, that contributes to the synthesis of additional myofibrils) [3]. The sequential expression of myogenic factors results in structural muscle protein gene expression, which includes myosin heavy chain gene (*MyHC*). Myosin is the main functional component of white muscle fibers, accounting for approximately 50% of all muscle proteins in fish [4]. The *MyHC* expression level can be used as an indicator reflecting the growth rate of muscle mass in fish and is necessary for assessing their growth and state in changing environmental conditions [5,6]. Negative regulators of muscle growth include myostatin, a member of the Transforming Growth Factor beta (TGFβ) superfamily, which is primarily expressed in skeletal muscle [7]. Its action is mediated by the inhibition of the activation, self-maintenance, and proliferation of myogenic progenitor cells (MPCs) [8]. Previous studies have shown that such environmental factors, as nutrition [9,10,11], temperature [12,13,14], environmental pH, oxygen availability [1], and photoperiod [15,16,17], can influence gene expression coding proteins that regulate myogenesis and muscle growth (i.e., MRFs) in fish. As a result, there is a change in the morphofunctional characteristics of skeletal muscle, which makes up the majority of the body in most fish species (~70%), that ensures the plasticity of fish growth.

The photoperiod is known to have a significant effect on fish [18], and extended daylight hours are widely used to stimulate the growth rate of salmonid fish species when cultured in hatchery and aquaculture conditions [19,20,21,22]. The photoperiod has been reported to affect the concentration of several hormones and growth factors that impact the behavior of MPCs [1]. It was found that the photoperiod affected differentiation of the muscle structure in *Umbrina cirrosa* fish larvae; hyperplasia was higher with a 24-h light regime, and hypertrophy was higher with a LD 16:8 regime [17]. In another study, it was shown that the introduction of continuous lighting during a period of natural decreasing day length led to an increase in the number of muscle fibers (hyperplasia) in Atlantic salmon and that this effect may have been enhanced or reduced in response to light exposure duration [15]. However, mechanisms that regulate the formation and growth of skeletal muscles in fish under the influence of light are yet to be fully elucidated. An assessment of factors that affect muscle protein gene expression is critical when rearing juvenile Atlantic salmon in fish farms in order to increase their vitality and readiness for release into a natural environment. 

In this regard, the objective of the current study was to evaluate the effect of continuous artificial lighting on the pattern of gene expression (*MyoD1* paralogs: *MyoD1a*, *MyoD1b*, *MyoD1c*; *Myf5*, *MyoG*, *MSTN* paralogs: *MSTN1a* and *MSTN1b*) that control muscle development and growth, as well as of the *MyHC* gene in the white muscles of Atlantic salmon (*Salmo salar* L.) juveniles (aged 1 year) reared in a fish hatchery during the year of the study. Understanding the molecular mechanisms underlying postembryonic muscle growth in fish would contribute to the development of biomarkers that could be used in genetic or ecological selection; such markers are an important tool for the effective management of fish growth.

## 2. Results

### 2.1. Weight Gain and Growth Rate

**Figure 1:**
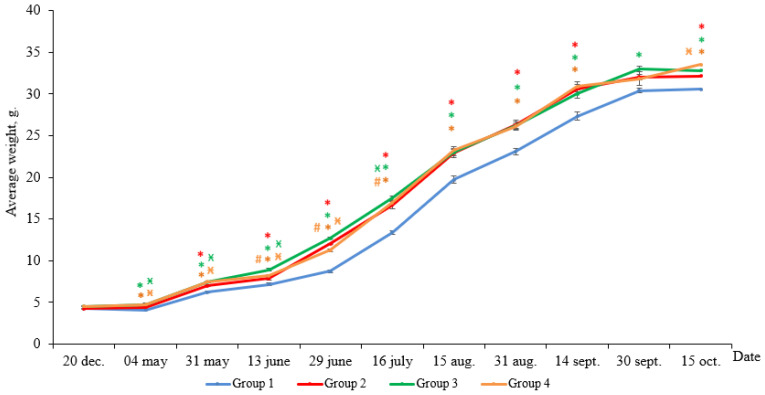
The average weight of the salmon yearlings reared under different light regimes (usual hatchery lighting versus constant lighting) between May and October (Group 1 (HL-hatchery lighting), Group 2 (LD 24:0 May–October 2018), Group 3 (LD 24:0 August–November 2017, May–October 2018), Group 4 (LD 24:0 August 2017–October 2018)). Vertical bars with the different superscript signs *^,^^ӿ,^^#^ represent a significant difference between groups (*p* < 0.05): *—significant differences compared with the HL group at the day of assessment, ^ӿ^—significant differences compared with Group 2 at the day of assessment, ^#^—significant differences compared with Group 3 at the day of assessment.

According to the results, the weight gain of yearlings at the end of the experimental period was 26.3 g in Group 1 (HL), 28.0 g in Group 2 (LD 24:0, May–October 2018), 28.1 g in Group 3 (LD 24:0, August–November 2017, May–October 2018), and 28.8 g in Group 4 (LD 24:0, August 2017–October 2018). At the end of the experiment, the average weight of the individuals in all Groups (2–4) with constant light significantly exceeded the weight of the fish in Group 1 (HL) (Figure 1, *p* < 0.05). Differences in the weight appeared in the first month of the study and remained until the end of the study.

**Figure 2:**
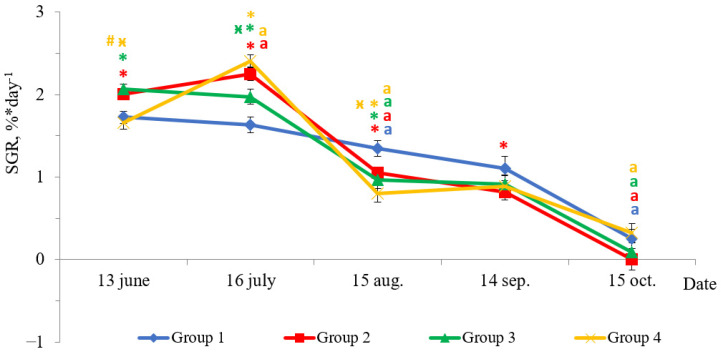
The specific growth rate (SGR, %* day^−1^) of salmon yearlings reared under different light regimes between May and October (Group 1 (HL-hatchery lighting), Group 2 (LD 24:0 May–October 2018), Group 3 (LD 24:0 August–November 2017, May–October 2018), and Group 4 (LD 24:0 August 2017–October 2018)). Vertical bars with the different superscript signs *^,^^ӿ^^, #^ and lowercase letter ^a^ represent a significant difference between groups (*p* < 0.05): *—significant differences compared with the HL group at the day of assessment, ^ӿ^—significant differences compared with Group 2 at the day of assessment, ^#^—significant differences compared with Group 3 at the day of assessment, ^a^—significant differences compared with the previous assessment day of the same group.

Fish reared under HL regime and continuous lighting differed by specific growth rate (SGR) during experimental period (Figure 2). In June, the SGR of fish in Groups 2 and 3 were higher than in Groups 1 (HL) and 4 (*p* < 0.05). The SGR of fish reared under continuous lighting (Groups 2, 3, and 4) were higher than in Group 1 (HL) in July (*p* < 0.05). In August, there was a sharp decline in SGR of fish in groups with LD 24:0 regime. The fish SGRs in these groups were 2–3 times less than those obtained in the previous month (Figure 2, *p* < 0.05), while the SGR of fish in Group 1 (HL) was 1.2 times less than the values in the previous month (Figure 2, *p* < 0.05). The SGR of fish in Group 1 (HL) were higher than in groups with additional lighting (*p* < 0.05) in August. The SGR of yearlings in all groups in September did not differ in comparison to the SGR of the previous period, while it decreased (*p* < 0.05) in October. The SGR of fish in Group 2 were lower than in HL Group (*p* < 0.05) in September.

### 2.2. Muscle-Specific Gene Expression

**Figure 3:**
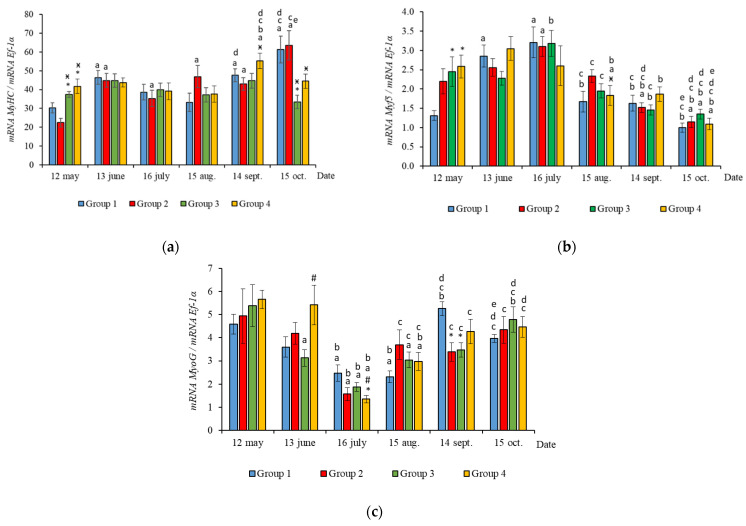
The relative expression of *MyHC* (**a**), *Myf5* (**b**), and *MyoG* (**c**) mRNA (units) in the white muscles of salmon yearlings reared according to exposure to different lighting regimes (HL—hatchery lighting, LD 24:0 (continuous lighting)). Values are means ± SE. Vertical bars with the different superscript signs *^,^^ӿ,^^#^ and lowercase letter ^a, b, c, d, e^ represent a significant difference between groups (*p* < 0.05): *—significant differences compared with the Group 1 (HL) at the day of assessment, ^ӿ^—significant differences compared with Group 2 (LD 24:0 May–October 2018) at the day of assessment, ^#^—significant differences compared with Group 3 (LD 24:0 August–November 2017, May–October 2018) at the day of assessment, ^a^—significant differences within the group compared with the data on 12 May, ^b^—significant differences within the group compared with the data on 13 June, ^c^—significant differences within the group compared with the data on 16 July, ^d^—significant differences within the group compared with the data on 15 August, ^e^—significant differences within the group compared with the data on 14 September.

**Figure 4:**
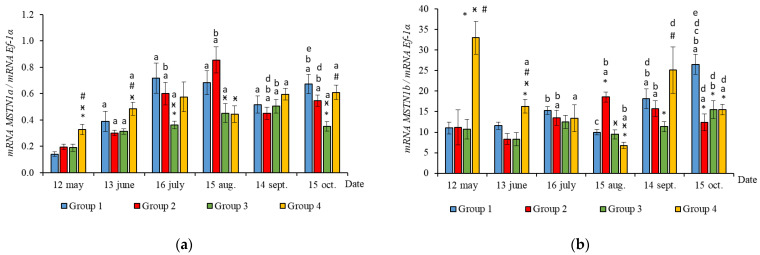
The relative expression of *MSTN1a* (**a**) and *MSTN1b* (**b**) mRNA (units) in the white muscles of salmon yearlings reared according to exposure to different lighting regimes (HL—hatchery lighting, LD 24:0 (continuous lighting)). Values are means ± SE. Vertical bars with the different superscript signs *^,^^ӿ,^^#^ and lowercase letter ^a, b, c, d, e^ represent a significant difference between groups (*p* < 0.05): *—significant differences compared with the Group 1 (HL) at the day of assessment, ^ӿ^—significant differences compared with Group 2 (LD 24:0 May–October 2018) at the day of assessment, ^#^—significant differences compared with Group 3 (LD 24:0 August–November 2017, May–October 2018) at the day of assessment, ^a^—significant differences within the group compared with the data on 12 May, ^b^—significant differences within the group compared with the data on 13 June, ^c^—significant differences within the group compared with the data on 16 July, ^d^—significant differences within the group compared with the data on 15 August, ^e^—significant differences within the group compared with the data on 14 September.

The RT-qPCR results showed seasonal variation in the expression levels of all genes studied during the experimental period and changes related to light regime in expression levels of several genes. *MyHC*, *MyoD1b*, *MyoD1c*, *MSTN1a,* and *MSTN1b* mRNA expression depended on the light regime used. A significantly higher level of *MyHC* mRNA expression (*p* < 0.050) was revealed in the white muscles of salmon in Groups 3 and 4 compared to the Groups 1 (HL) and 2 in May (Figure 3a, *p* < 0.05). The expression of *Myf5* was significantly higher (*p* < 0.050) in Groups 3 and 4 compared to Group 1 (HL) in the same month (Figure 3b, *p* < 0.05). In addition, the expression of *MSTN1a* and *MSTN1b* mRNA was higher in salmon in Group 4 compared to the Groups 1 (HL), 2, and 3 in the beginning of the study (Figure 4, *p* < 0.05).

**Figure 5:**
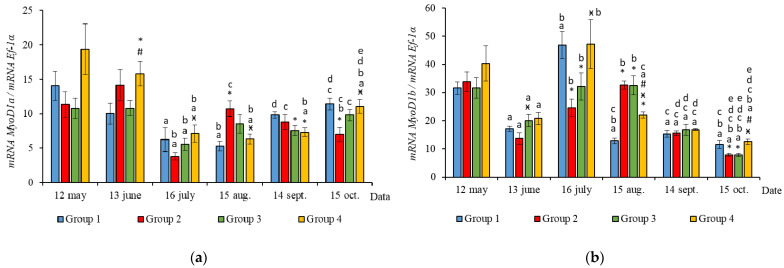

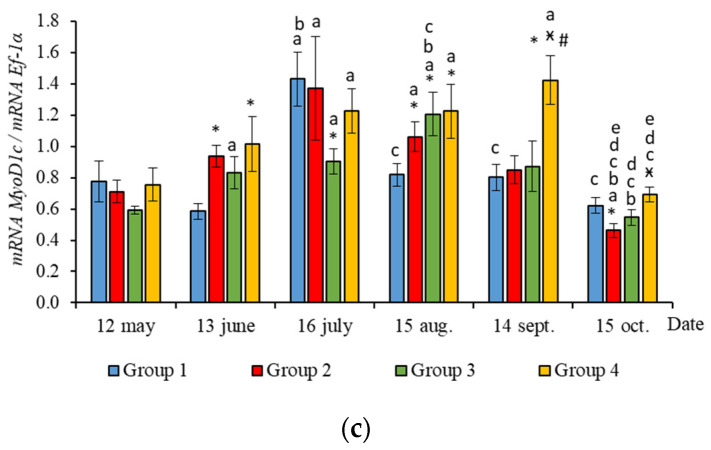
The relative expression of *MyoD1a* (**a**)**,** *MyoD1b* (**b**), and MyoD1c (**c**) mRNA (units) in the white muscles of salmon yearlings reared according to exposure to different lighting regimes (HL—hatchery lighting, LD 24:0 (continuous lighting)). Values are means ± SE. Vertical bars with the different superscript signs *^,^^ӿ,^^#^ and lowercase letter ^a, b, c, d, e^ represent a significant difference between groups (*p* < 0.05): *—significant differences compared with the Group 1 (HL) at the day of assessment, ^ӿ^—significant differences compared with Group 2 (LD 24:0 May–October 2018) at the day of assessment, ^#^—significant differences compared with Group 3 (LD 24:0 August–November 2017, May–October 2018) at the day of assessment, ^a^—significant differences within the group compared with the data on 12 May, ^b^—significant differences within the group compared with the data on 13 June, ^c^—significant differences within the group compared with the data on 16 July, ^d^—significant differences within the group compared with the data on 15 August, ^e^—significant differences within the group compared with the data on 14 September.

In June, the yearlings in Group 4 had the highest mRNA expression values for *MyoG* in comparison to Group 3 (Figure 3c, *p* < 0.050), as well as the highest *MyoD1a* expression in comparison to Groups 1 and 3 (*p* < 0.050), *MyoD1c* in comparison to the Group 1(HL) (*p* < 0.050) (Figure 5a,c), *MSTN1b* expression in comparison to the other groups, and *MSTN1a* expression in comparison to Groups 2 and 3, *p* < 0.050 (Figure 4). The *MyoD1c* expression levels were higher in Group 2 than in the Group 1 (HL) (Figure 5c).

In July, the levels of mRNA expression of the *MyoD1b, MyoD1c* (Figure 5b,c, *p* < 0.050)*,* and *MSTN1a* (Figure 4a, *p* < 0.05) paralogs were higher in fish in the Group 1 (HL) compared to Group 3. In addition, the expression levels of the *MyoD1a* and *MyoD1b* paralogs in fish in Group 4 significantly exceeded those in fish in Group 2 (Figure 5a,b).

In August, the highest values for *Myf5, MSTN1b, MSTN1a, MyoD1a,* and *MyoD1b* gene expression were observed in individuals in the Group 2 (Figure 3b, Figure 4 and Figure 5a,b). The mRNA *MyoD1c* and *MyoD1b* expression levels in fish in the Group 1 (HL) were lower than those in the groups exposed to additional lighting (Figure 5b,c, *p* < 0.05).

In September, significantly higher levels of *MyHC* (in comparison to Group 2), *MSTN1b* (in comparison to Group 3) and *MyoD1c* (in comparison to all groups) expression were observed in fish in Group 4 (Figure 3a, Figure 4b and Figure 5c, *p* < 0.05). The expression of *MyoG* and *MyoD1a* was higher in fish in the Group 1 (HL) compared to the groups reared under continuous lighting (Figure 3a, Figure 4b and Figure 5c, *p* < 0.05).

On completion of the study, the expression values of the *MSTN1b* and *MyoD* paralogs in the white muscle of fish in the Group 1 (HL) significantly exceeded those of fish in Group 2 (Figure 4b and Figure 5, *p* < 0.05), while the expression levels of the *MyHC, MyoD1b,* and *MSTN* paralogs also exceeded those of Group 3 (Figure 3a, Figure 4 and Figure 5b, *p* < 0.05).

In general, in all studied groups in July, there was a noticeable decrease in the levels of *MyoG* and *MyoD1a* mRNA expression (Figure 3c and Figure 5a, *p* < 0.05), along with a gradual increase in these parameters by the end of the experiment in autumn.

Yearlings in Groups 2, 3, and 4 were characterized by a gradual decrease in the *Myf5* and *MyoD1b* gene expression levels from August to October (Figure 3b and Figure 5b). In salmon in the Group 1 (HL), expression of these genes began to decrease in July (Figure 3b and Figure 5b). Similarly, in Groups 1 (HL), 2, and 3, this was accompanied with a decrease in the level of *MyoD1c* expression (Figure 5c). The level of *MyoD1c* expression in Group 4 remained at the same level until September and decreased by mid-October (Figure 5c). 

### 2.3. The Relationship with the Growth Rate (SGR)

A positive relationship was established between the SGR of yearlings and the level of *MyoD1b* expression in Groups 1 (HL) and 4 (Table 1, *p* < 0.05), and between the SGR and the level of *MyoD1c* expression in the Group 1 (HL) (Table 1, *p* < 0.05). The relationship between *Myf5* expression and SGR was positive in all the Groups exposed to additional lighting (Table 1, *p* < 0.05).

### 2.4. Correlation Analysis

The results of the correlation analysis of gene expression are presented in Table 2, Table 3, Table 4 and Table 5. A positive correlation was demonstrated between *Myf5* and *MyoD1b* expression and between *MyoG* and *MyoD1a* expression in all groups studied (*p* < 0.05). *MyHC* was positively correlated with *MyoD1a* expression in the Groups 1 (HL) and 3 and with *MyoG* in Groups 2 and 4.

In Groups 1 (HL), 2, and 3, a positive correlation was observed between the two paralogs, *MyoD1b* and *MyoD1c* (Table 2, Table 3 and Table 4, *p* < 0.05), while in Group 4, levels of *MyoD1b* expression were positively associated with *MyoD1a* (Table 5, *p* < 0.05).

In Groups 1 (HL) and 2, the expression levels of *MSTN1b* were positively linked to *MSTN1a* expression (Table 2 and Table 3, *p* < 0.05). In Groups 1 (HL), 2, and 3, *MyoD1c* expression levels were positively related to *MSTN1a* expression (Table 2, Table 3 and Table 4, *p* < 0.05). *MyHC* expression was positively associated with *MSTN1a* expression in the experimental groups (Groups 2–4) (Table 3, Table 4 and Table 5, *p* < 0.05). In addition, *MyHC* expression levels had a positive correlation with *MSTN1b* expression in Groups 1 (HL), 2, and 4 (Table 2, Table 3 and Table 5, *p* < 0.05). *MyoG* positively correlated with *MSTN1b* in Groups 3 and 4 (Table 3 and Table 5, *p* < 0.05) and with *MSTN1a* in the Group 1 (HL) (Table 2, *p* < 0.05).

## 3. Discussion

### 3.1. Weight Gain and Growth Rate

A significantly higher body weight gain was demonstrated in fish reared under continuous lighting, compared to the Group 1 (HL), throughout the experiment. The finding in the current study of the enhanced growth of juvenile salmon due to artificial lighting is consistent with the results of previous studies obtained on Atlantic salmon parr [23,24] and juvenile rainbow trout reared for an extended photoperiod in fresh water [19,20]. It has been suggested that exposure to constant lighting could directly stimulate growth in rainbow trout [15]. Handeland et al. [25] showed that the use of constant light promoted growth acceleration in Atlantic salmon by stimulating food intake. Increased food intake could also compensate for the high metabolic rate and greater locomotor activity of fish under constant light conditions [26,27]. Although the fish are exposed for more daylight hours in which they feed, the increase in their growth is most likely to be due to physiological changes that lead to an improved appetite and/or feed conversion rate (FCR) [28,29]. Thus, Nordgarden et al. [30] found a clear seasonal pattern of growth and feed intake and showed that an increase in their growth under constant lighting conditions was associated with an improvement in the FCR and an increased appetite in Atlantic salmon. Further research is necessary to control for such physiological parameters as FCR and fish feed intake, to clearly determine the mechanisms responsible for improvement in the growth of fish exposed to constant light or any manipulations with light regime. It is likely that short-term exposure to the photoperiod induces metabolic changes in fish, which, in turn, promotes higher growth rates due to the more efficient use of nutrients [16]. These processes in fish are controlled by growth hormone, which is produced in the pituitary gland in response to changes in the length of day [31,32]. Growth hormone plays an important physiological role in the metabolism of proteins, carbohydrates, and lipids; it also stimulates skeletal growth and the growth of skeletal muscles in teleost fish, by affecting the expression of several genes, including MRFs and myostatin [33,34]. The photoperiod is probably the most important factor that determines the seasonal pattern of somatic growth in salmonids through regulation of the neuroendocrine system of fish [35,36].

Fish in Group 4 (LD 24:0 August 2017–October 2018), reared under constant light, even in winter, were observed to weigh much more than fish in the Groups 1 (HL), 2, and 3 upon completion of the study. This finding is consistent with the data obtained in a study on Atlantic salmon [15], where constant light during the winter–spring period improved the growth performance of the fish and resulted in an increase (of 30%) in their mean body weight after 126 days of exposure, compared to fish exposed to natural lighting.

Notably, fish reared under the HL regime and under continuous lighting differed by SGR during the experimental period. In particular, the differences were significant during summer. The SGR of fish reared under continuous lighting were higher than the SGR of fish reared in the HL group in July. However, in August, a sharp decrease in the SGR of fish in groups exposed to LD 24:0 regime was observed, when compared to the SGR of fish in the HL group. Although the SGR also decreased in Group 1 (HL), the corresponding values were not as low as those observed in Groups 2–4. It is probable that this was as a result of an increase in water temperature (up to 21 °C) in July and August and related with restriction in rations.

It is necessary to note that in our previous study [37], salmon yearlings exposed to experimental regimes (LD 16:8 and LD 24:0 between July and October) experienced weight loss during the first 2 months, but the growth rate of fish in the HL group decreased in September and October, and yearlings in the experimental groups continued to grow. It has been suggested that yearlings need a period to adapt to new light conditions. In the current study, we did not observe this. The additional lighting provided in May was more favorable and closer to natural lighting as it was added in a period characterized by an increase in the number of daylight hours.

### 3.2. Muscle-Specific Gene mRNA Expression

The expression levels of the studied genes varied throughout the study and were dependent on the season. *MyHC, MyoD1c, MyoD1b,* and *MSTN1a/b* mRNA expression was influenced by the light regime used. Significantly higher values of *MyHC* mRNA expression were observed in May in fish exposed to continuous lighting in Groups 3 and 4, compared to fish in Groups 1 (HL) and 2. Previously, it was shown that *MyHC* mRNA expression correlated with weight [38], as well as with the growth rate in Atlantic salmon [5]. In our work, the data for *MyHC* mRNA expression were consistent with differences in the weight of the fish during this period. This can perhaps be explained by the fact that the salmon yearlings in Groups 3 and 4 were underyearlings in the study conducted the previous year and grew under 24-h lighting for 3 months. Along with the high expression of *MyHC*, fish in Groups 3 and 4 had significantly higher levels of *Myf5* mRNA expression at the start of the study when compared with the HL group. Transcription factors are known to sequentially regulate the stages of myogenesis, i.e., *MyoD* and *Myf5* are required for myoblast determination and *MyoG* is involved in their differentiation and fusion with the formation of myofibers [2]. Their expression depends on many factors and determines the processes of muscle fiber hyperplasia and hypertrophy [1,11,13,15]. Studies on Atlantic salmon and rainbow trout have shown that earlier exposure to constant light had a substantial effect on muscle hyperplasia in fish, thereby promoting the formation of new muscle fibers, and resulted in significantly greater weight gain of fish [15,20]. In addition, as soon as the hyperplasia process ceased, muscle growth occurred due to hypertrophy of the previously formed fibers that led to significant increase in body weight and condition factor of fish reared under constant light [15,39]. The final body weight of fish depends on hypertrophy and muscle hyperplasia. It is known that the hyperplastic process of muscle growth relates directly to a proliferation (increase in the population) of undifferentiated MPCs, which express the primary MRFs, *Myf5* and *MyoD* [2]. Previously, in a study on juvenile Atlantic cod, compared to fish that grew under natural photoperiod conditions, it was established that *Myf5* expression was significantly higher in the group exposed to continuous lighting after just 12 h and again 30 days after the start of the experiment. In addition, these fish were approximately 13% and 11% larger on study days 120 and 180, correspondingly [16].

Subsequently, in June, the salmon yearlings in Group 4 had significantly higher mRNA expression levels of *MyoD* paralogs (*MyoD1a*, compared to Groups 1 (HL) and 3; *MyoD1c*, compared to Groups 1 (HL)), as well as *MyoG* expression (compared to Group 3). *MyoD1* is characterized by three paralogs that subfunctionalize and exhibit distinct expression patterns in different fiber types and throughout development [40]. It was shown in salmon primary cell cultures that *MyoD1* paralogs were differentially expressed in the proliferating and differentiating cells during myotube maturation, and it was suggested that *MyoD1b* and *MyoD1c* regulate the cell cycle and *MyoD1a* is involved in terminal differentiation [41]. As previously shown, high levels of *MyoD* mRNA expression in juvenile fish were associated with a predominant hyperplastic mechanism of muscle growth, while myogenin expression was linked to myoblast differentiation and hypertrophy [3,42]. Nagasawa et al. [16] found that an increase in the growth of juvenile Atlantic cod under continuous lighting was accompanied by the increased expression of *pax7, Myf5,* and *MyoG*. Thus, it is likely that the differences observed in the mRNA levels of *Myf5, MyoD1,* and *MyoG* paralogs in fish from the different experimental groups in the current study reflect differences in the regulation of hyperplastic and hypertrophic muscle growth processes caused by the additional lighting. Notably, higher *MyoD1c* mRNA expression values were observed in Group 2 (first exposed to additional lighting in May), compared to the HL group, just a month after this exposure. The differential expression of MRFs in the muscle of salmon in tanks with different light regimes may indicate variations in fish growth patterns.

According to the data for the entire study period, differences were observed in the relationship between SGR and MRFs in groups of fish with different light regimes. The SGR was positively correlated with *Myf5* in fish in tanks with continuous lighting, while a positive correlation between the SGR and *MyoD1c* expression levels was established in fish in the Group 1 (HL). In addition, the SGR positively correlated with *MyoD1b* expression levels in fish in the Group 1 (HL) and Group 4. Thus, the positive correlation between *MyoD1b/1c* and *Myf5* and the SGR was shown to depend on the lighting regime, which probably indicates a relationship between the regulation of myoblast determination and proliferation processes and the growth rates of the salmon yearlings throughout the study. Fish growth is known to correlate with muscle development and depends on environmental conditions that impact the rate of myogenesis, cell organelle composition, gene expression patterns, and the number and size distribution of the muscle fibers [43]. *Myf5* and *MyoD1c* have been demonstrated to be promising candidate genes in the cellular-level signaling system that regulates fish myotomal muscle growth, since they are highly expressed in salmon muscles in response to refeeding [44].

According to the results, general trends in seasonal variations of gene expression were established in salmon yearlings in tanks exposed to different lighting regimes throughout the study. A gradual decrease in the expression levels of *Myf5* and *MyoD1b* genes from August to October was characteristic of fish in groups with additional lighting (Groups 2–4), and this was consistent with a decrease in the rate of fish growth in autumn, whereas it was associated with a decrease in the levels of *MyoD1c* expression in Groups 2 and 3. In fish in Group 4, the *MyoD1c* mRNA expression remained at a high level throughout the 4 months of the study (June to September), decreasing only in mid-October. A decrease in the level of expression of the above genes in fish in the Group 1 (HL) was observed a month earlier, in the period from July to October. It should be noted that *MyoD1c* and *MyoD1b* mRNA expression levels in fish in the experimental groups exposed to additional lighting were higher than those in fish in the Group 1 (HL) in August, and this was also associated with the earlier decrease in these parameters in the Group 1 (HL). It could also indicate differences in the mechanisms of muscle growth regulation in fish in relation to environmental conditions (light and water temperature in different months in the study). In addition, a positive correlation was observed between the expression levels of *Myf5* and *MyoD1b* in all groups. A similar relationship between the expression levels of these genes was shown during the maturation of primary myogenic culture derived from the fast skeletal muscle of Atlantic salmon [41]. According to statistical analysis, the expression levels of *MyoD1b* and *MyoD1c* were dependent on the lighting regime, and changes in this regard were also associated with seasonality, while the expression of *Myf5* in fish in tanks with different lighting was only dependent on the season. It is possible that the simultaneous decrease in *MyoD1b, Myf5,* and *MyoD1c* mRNA expression in all groups with different light regimes was associated with changes in the mechanisms of muscle growth in fish. In nature, the growth of fish, such as Atlantic salmon, in temperate latitudes is cyclical since seasonal changes in photoperiods and temperatures can contribute to periods of faster (i.e., spring and summer growth profiles) or slower growth, accompanied by a decrease in the water temperature and daylight hours (autumn and winter periods) [45]. Since the photoperiod was controlled in the current study, and the temperature regime was natural, differences in the expression of MRFs were probably attributable to seasonal patterns of fish growth. Johnston et al. [15] suggested that there is a seasonal cycle of muscle formation that superimposes on the endogenous growth rhythm associated with age and/or the developmental stage of fish. Temperature affects muscle growth patterns in fish, modulating the rates of muscle fiber hypertrophy and hyperplasia by influencing the signaling pathways that regulate the proliferation and differentiation of MPCs, protein synthesis and degradation, and gene expression pattern [1,13,15,46]. Elsewhere, it was shown in a study on the larvae of sea bass (*Dicentrarchus labrax L*.) that muscle fiber hypertrophy was greater in fish reared at an ambient temperature (approximately 15 °C), while the proliferation of new muscle fibers (hyperplasia) was greater at 19 °C [47]. In the current study, the water temperature varied between mid-July and mid-August in range of 19–22 °C, gradually decreasing to 15 °C by mid-September. We hypothesized that a joint decrease in the mRNA expression of *Myf5* and *MyoD* paralogs (*MyoD1b* and *MyoD1c*) is associated with a decrease in myoblast activation, that probably leads to a decrease in muscle growth through muscle fiber hyperplasia and a transition to the hypertrophic growth mechanism caused by changes in the temperature conditions of the fish habitat.

This observation is consistent with the data on the mRNA expression of *MyoG* and *MyoD1a* in the same period (August to October). Thus, there was a simultaneous increase in the mRNA expression levels of *MyoG* and *MyoD1a* in Groups 1 (HL), 3, and 4. *MyoG* mRNA expression levels were seen to positively correlate with the *MyoD1a* expression levels in all groups. In other research on myoblast cell culture, it was also shown that *MyoG* expression strongly correlated with *MyoD1a* expression [41]. It was reported that *MyoD* activates *MyoG* expression by directly binding to its regulatory elements [48]. In particular, *MyoD1a* is involved in the terminal differentiation of myoblasts into myocytes [41], and myogenin expression is associated with both terminal differentiation and the subsequent maturation and hypertrophy of myofibrils [3,42]. In our work, the expression level of these genes depended only on water temperature, as a seasonal factor. The expression levels of these genes were significantly higher for all fish in the studied groups on completion of the study (mid-October), compared to the levels observed in mid-July when the water temperature was elevated. This can reflect the previously mentioned regularity of changes to interactions between hypertrophic and hyperplastic muscle growth processes in response to water temperature [47]. In addition, a consistent increase in *MyoD* and *MyoG* mRNA expression can also reflect features of the simultaneous expression of these genes associated with the age or developmental stage of the fish, as previously shown in Atlantic salmon [49], rainbow trout (*Oncorhynchus mykiss*) [3], and pacu (*Piaractus mesopotamicus*) [50].

A positive correlation between the mRNA expression levels of MRFs and *MyHC* (between *MyHC* and *MyoD1a* in Groups 1 (HL) and 3 and between *MyHC* and *MyoG* in Groups 2 and 4) was observed throughout the study. Importantly, the expression of the *MyHC* gene increased by mid-October, reaching a maximum in Groups 1 (HL) and 2, while it remained at approximately the same levels throughout the study in Groups 3 and 4. A similar result was obtained by us earlier in a study on salmon underyearlings (0 +) in which fish in all groups (with different photoperiod regimes) experienced a joint increase in *MyHC, MyoD1a,* and *MyoG* mRNA expression levels by the end of October [51]. It has been suggested that an increase in the mRNA expression of *MyHC* occurs in response to a decrease in water temperature and is associated with the necessity of increasing *MyHC* gene transcripts, which have a greater ATPase activity, as has been shown previously in the case of carp [52] and channel catfish [53]. 

Thus, the mRNA expression levels of *MyoD1a* and *MyoG* in fish in all the groups increased by mid-October, when compared to July levels; conversely, the mRNA levels of *MyoD1b, Myf5,* and *MyoD1c* decreased. This result is consistent with the data obtained in our previous study that evaluated the effect of different photoperiod regimes on Atlantic salmon underyearlings [51]. This can be an evidence of seasonal changes in the expression of MRFs associated with a decrease in water temperature. It can be assumed that there is a decrease in myoblast determination processes and an increase in the regulation of the muscle cells terminal differentiation, thus leading to an increase in *MyHC* expression.

A significant correlation between the expression levels of MRFs and *MSTN* paralogs (*MSTN1a* and *MSTN1b*) was observed in all the studied groups of salmon yearlings throughout the study. The expression levels of *MyoG* positively correlated with the level of *MSTN1b* in fish in Groups 3 and 4. A similar positive correlation was observed between the mRNA expression levels of *MyoG* and *MSTN* genes in trout [3]. The mRNA expression levels of *MyoD1c* positively correlated with level of *MSTN1a* in fish in Groups 1 (HL), 2, and 3. In addition, *MyoD1b* mRNA correlated with the expression levels of *MSTN1b* in fish in Groups 2 and 4. *MSTN* is known to inhibit the proliferation and differentiation of myoblasts and cease the division of MPCs [54,55]. It is probable that *MyoG* is one of the main physiological targets of *MSTN* [56,57]. *MyoD* binding sites have also been found in the *MSTN* promoter [57]. Differences in the functioning of distinct *MSTN* paralogs and their role in the regulation of myogenesis remain unclear. A positive correlation between *MyHC* mRNA and *MSTN1a* was observed in all the experimental fish groups exposed to additional lighting. *MyHC* mRNA level correlated with the *MSTN1b* expression in Groups 1 (HL), 2, and 4 as well. In addition, the mRNA expression levels of *MSTN1a* were seen to have a positive correlation with the fish weight in all the groups. A possible explanation for this is that *MSTN* paralogs were expressed in response to the elevated expression of *MyHC* and *MRFs*, that is a necessary regulatory mechanism for attenuating muscle fiber hyperplasia and hypertrophy and, as a consequence, controlling muscle growth [3,58].

## 4. Materials and Methods

### 4.1. Experimental Design

**Figure 6:**
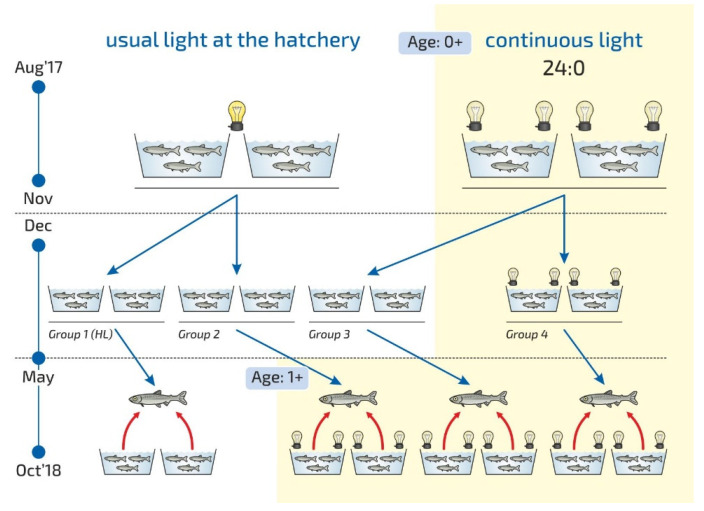
Scheme of the experiment at the Vygsky fish hatchery. Different photoperiod regimes: usual light at the hatchery, HL (hatchery lighting); the experimental regimes—continuous light, LD 24:0—are described in detail in the text.

The research was carried out at the Vygsky Fish Hatchery, Belomorskij Region, Russia (64°25′ N and 34°28′ E). The study period was 6 months (May–October 2018). The effects of the LD 24:0 light regime on the growth and condition of yearlings were studied. The study is a continuation of previous research on the influence of light regimes on the growth of juvenile fish, which was conducted in 2017 [37]. We continued to monitor fish that grew in the first year in tanks without additional lighting (the control group) and those under the LD 24:0 regime (the experimental group) during August–November 2017 (Figure 6). In December 2017, medium-weight underyearlings from the control tanks (4.2 ± 0.04 g) and those from the tanks exposed to constant lighting (4.5 ± 0.04 g) were transferred to new tanks (1054 fish per tank) for overwinter period. The experimental fish were placed in four groups according to the different photoperiod regimes started in May 2018. Two parallel tanks were used for each group regime (Figure 6). The first and second groups comprised fish, which had been reared in usual hatchery light conditions without additional lighting in the previous 2017 year. (1) Additional light was not turned on in Group 1 (Group 1 (HL), Hatchery lighting). (2) The second group was reared under continuous lighting from May–October 2018 (Group 2, LD 24:0 May–October 2018). The third and fourth groups comprised fish exposed to continuous light between August and November 2017. (3) The third group was reared under continuous light from May–October 2018 (Group 3, LD 24:0 August–November 2017, May–October 2018). (4) The fourth group was exposed to constant light during the whole period of the study during August 2017 to October 2018 (Group 4, LD 24:0, August 2017–October 2018). The effect of a continuous lighting (Groups 2, 3, 4) were compared with the usual hatchery lighting regime (Group 1 (HL)).

**Figure 7:**
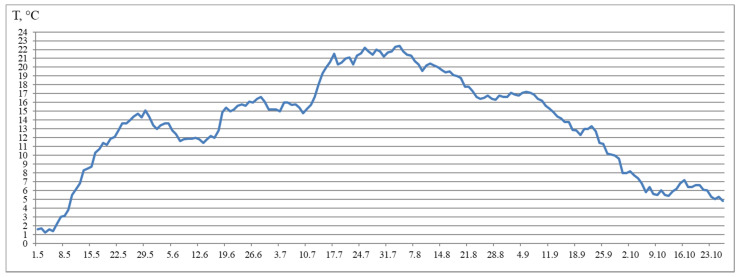
Water temperature from May–October.

The fish were kept in flow in tanks (4 m^2^) and with a water volume of 720 L. The experimental tanks (for groups 2–4) were equipped with two light-emitting diodes (LED) lights (Aquael Leddy^®^ Smart LED Sunny, 6 W, 6500 K, Aquael, Warsaw, Poland) in May 2018, placed in the tank wells diagonally across from each other and covered with black, light-tight film. The light intensity was 760 lx at the water surface under the LED lights and 400 lx around it, 45 lx at the opposite side, and 70 lx at the center of the tank. The usual light regime of the hatchery was used for Group 1. The Group 1 (HL) was subjected to natural light from outside from May to the half of August 2018, and the light intensity at the water surface was measured to be 12–29 lx during daylight hours and 2–4 lx at night. Sunrise and sunset at the location of the Vygsky fish hatchery and certain latitude (64°25′ N and 34°28′ E) can be found here: https://sunsetsunrisetime.com/sun/belomorsk (accessed on 9 April 2021). Then, the hatchery light was turned on from 5 p.m. to 8 a.m. for a month. The lamps were located on the building walls and were positioned sideways. The light intensity at the water surface in the center of the tank was 10 lx in daylight hours and 8 lx at night. The hatchery lighting was turned on continuously starting on 10 September 2018. The light intensity at the water surface was uneven (8 lx at the center). All other rearing conditions, such as fish-holding density, feed and feeding regime, preventive measures, and care of the tanks, remained the same. The fish were fed with commercial feed according to recommendations of the fish hatchery, and this depended on water temperature fluctuations. For the yearlings, commercial feed (BioMarInicio^®^ 917, BioMar, Denmark), which contained 47–50% crude protein, 16–23% crude lipids, and 15–20% carbohydrates, depending on pellet size, was used. The daily rations for yearlings were 0.1–0.8% and 1.3–1.5% in May, 1.8–1.9% in June, 1.8–1.5% in July, 1.3–2.0% in August, 1.2–0.9% in September, and 0.5% in October. Automatic feeders were used to feed yearlings. The feeding regime was organized according to the general rules and recommendations of the hatchery and based on water temperature (this factor influences active fish growth duration) and the sustainable feeding practices relating to hatchery-reared fish. Water flowed into the tanks from the Matkozhnenskoe Reservoir (Nizhnij Vyg River) (flow velocity of 60 L min^−1^) using a natural temperature regime (Figure 7).

At the beginning of the study in May 2018, the fish in each tank (160 fish/per tank) were tagged using PIT tags (Felixcan^®^ SL, Felixcan, Spain). The PIT-tag injection procedure was as follows: the fish were anesthetized with clove oil, and medium-sized individuals (weighing 4–5 g) were tagged. The average weight of the fish in the groups at the beginning of the study in May was 4.13 ± 0.03 g (Group 1 (HL)), 4.20 ± 0.03 g (Group 2, LD 24:0, May–October 2018), 4.62 ± 0.04 ± 0.03 g (Group 3, LD 24:0, August–November 2017, May–October 2018), and 4.61 ± 0.04 (Group 4, LD 24:0, LD 24:0, August 2017–October 2018). Every month, weight and length of the 40 tagged fish per group were measured. The weighting data for the tagged fish were used to determine the specific growth rate (SGR), calculated as SGR (% × day^−1^) = [(ln final weight−ln initial weight)/n days] × 100. In parallel, weighting of the random fish in the tanks was conducted 3 times a month—the data on fish weight were obtained by repeatedly weighing (5 times) of 100 individuals together. These data were used to describe the weight gain of the fish.

The epaxial (white) muscle samples for real-time PCR (n = 10 per group) were selected at the beginning of the study and thereafter on a monthly basis (12 May, 13 June, 16 July, 15 August, 14 September, and 15 October 2018). The samples were frozen in liquid nitrogen and stored at −80 °C before the analysis. A description of the fish taken for analysis is provided in Table 6.

### 4.2. Muscle-Specific Gene mRNA Expression

Total RNA was isolated from epaxial muscle samples using an RNA-extra kit an analogue of a TRIzol^®^ RNA extraction kit (Evrogen, Moscow, Russia) as per the manufacturer’s protocol. Total RNA was then treated with DNase (Sileks, Moscow, Russia). RNA integrity and quality were assessed using 1% agarose gel electrophoresis and spectrophotometrically at an 260/280 nm absorbance ratio (Implen NanoPhotometer^®^ C, Implen, Germany). The RNA was reverse transcribed using MMLV-Reverse Transcriptase^®^ and random hexamer primers (Evrogen, Russia).

A real-time PCR assay was conducted using the CFX96^®^ Touch Real-Time Protection PCR System (BioRad, Hercules, CA, USA). The primers for the fast skeletal *MyHC*, myogenin (*MyoG*), *MyoD1a*, *MyoD1b*, *MyoD1c*, *Myf5*, *MSTN1a*, *MSTN1b,* and elongation factor-1 (*Ef-1α*) were selected using Beacon Designer^®^ 5.0 software (Premier Biosoft, San Francisco, CA, USA). The primer sequences are provided in Table 7. Amplification of 2 µL of cDNA (1:5 dilution of RT reaction) occurred using 5 µL of qPCRmix-HS SYBR Green^®^ 5x (Evrogen, Russia) and 500 nM primers, which yielded a final volume of 25 µL. The real-time conditions were as follows: DNA denaturation for 5 min at 95 °C, repeating cycles (40) of denaturation for 20 s at 95 °C, annealing for 30 s at 60 °C, and DNA elongation for 30 s at 72 °C. The specificity of the qPCR reaction and the presence of primer dimers were determined from the melting curves generated under the dissociation protocol (between 65 and 97 °C). Standard curves corresponding to a fivefold dilution series of mixed cDNA from all the samples facilitated a calculation of PCR efficiency. Each sample was run in triplicate on a single plate. The relative expression levels of the genes were determined by the threshold cycle (Ct) method and normalized against the *Ef-1α* using the 2^−ΔCt^ method [59].

### 4.3. Statistical Analysis

The data were tested for normality of variance using the Shapiro–Wilk test. A two-way ANOVA test with the light regime and date of sampling (season) as the factors was applied. Tukey’s post hoc test was conducted. The Pearson correlation coefficient was used to evaluate the relationship between the values of gene expression. Multiple regression analysis and the Pearson correlation coefficient were used to examine the relationship between the values of gene expression and SGR. A finding of *p* < 0.050 was considered as statistical significance. The data were presented as the mean ± SE.

## 5. Conclusions

Based on the study’s findings, the introduction of continuous lighting had a positive effect on the growth of juvenile Atlantic salmon. A substantial increase in the weight of fish reared under continuous light for 16 months (also in winter) was observed in relation to the other groups upon completion of the study. The features of muscle protein gene expression in Atlantic salmon yearlings were associated with the rearing conditions of juveniles, which included such factors as the lighting regime and temperature. It was shown that there are certain seasonal patterns of the simultaneous expression of MRFs both in fish reared under continuous exposure to light and in the HL group. We hypothesized that this could be elucidated by changes in the ratio of hyperplastic to hypertrophic muscle growth processes associated to seasonal temperature.

It was established that the expression of *MyoD1* paralogs changed in different ways throughout the study and was probably defined by their origin and, consequently, functional differences in regulation of myogenesis. In all the studied groups, a significant correlation was found between the expression levels of MRFs and *MSTN* paralogs throughout the study.

In future studies, when examining the molecular mechanisms that underlie plasticity of muscle growth in Atlantic salmon under light stimulation, histological analysis of muscle fibers would also be warranted to confirm the physiological mechanisms that promote growth.

## Figures and Tables

**Table 1:** Linear regression and coefficients of correlation between gene expression levels of the MRFs, *MyHC,* and *MSTN* paralogs in the white muscles of salmon yearlings and the SGR of salmon yearlings in groups exposed to different lighting regimes (Group 1—HL—hatchery lighting; Group 2—LD 24:0, May–October 2018; Group 3—LD 24:0, August–November 2017, May–October 2018; Group 4—LD 24:0, August 2017–October 2018).

Variable (y)	Group	SGR (x)	Coefficient of Correlation (SGR)
*MyHC*	1	y = 51.94 − 4.76x R^2^ = 0.057	−0.24
	2	y = 60.53 − 10.66x R^2^ = 0.378 *	−0.61 *
	3	y = 38.36 + 1.42x R^2^ = 0.014	0.12
	4	y = 46.12 − 1.53x R^2^ = 0.017	−0.13
*MyoG*	1	y = 3.98 − 0.35x R^2^ = 0.036	−0.19
	2	y = 4.26 − 0.67x R^2^ = 0.138 *	−0.37 *
	3	y = 4.09 − 0.71x R^2^ = 0.246 *	−0.50 *
	4	y = 4.29 − 0.47x R^2^ = 0.056	−0.24
*Myf5*	1	y = 1.60 + 0.36x R^2^ = 0.061	0.25
	2	y = 1.37 + 0.65x R^2^ = 0.470 *	0.69 *
	3	y = 1.52 + 0.42x R^2^ = 0.207 *	0.45 *
	4	y = 1.46 + 0.45x R^2^ = 0.184 *	0.43 *
*MyoD1a*	1	y = 10.23 − 1.35x R^2^ = 0.086	−0.29
	2	y = 9.07 − 0.30x R^2^ = 0.004	−0.06
	3	y = 8.90 − 0.34x R^2^ = 0.010	−0.10
	4	y = 9.23 + 0.05x R^2^ = 0.00	0.01
*MyoD1b*	1	y = 9.78 + 7.78x R^2^ = 0.178 *	0.42 *
	2	y = 14.75 + 3.44x R^2^ = 0.104	0.32
	3	y = 17.89 + 3.39x R^2^ = 0.069	0.26
	4	y = 14.27 + 7.50x R^2^ = 0.227 *	0.48 *
*MyoD1c*	1	y = 0.63 + 0.17x R^2^ = 0.109 *	0.33 *
	2	y = 0.72 + 0.17x R^2^ = 0.089	0.30
	3	y = 0.82 + 0.04x R^2^ = 0.011	0.10
	4	y = 1.10 + 0.01x R^2^ = 0.001	0.03
*MSTN1a*	1	y = 0.66 − 0.05x R^2^ = 0.027	−0.16
	2	y = 0.63 − 0.06x R^2^ = 0.041	−0.20
	3	y = 0.47 − 0.05x R^2^ = 0.092	−0.30
	4	y = 0.53 + 0.00x R^2^ = 0.001	0.02
*MSTN1b*	1	y = 22.37 − 5.04x R^2^ = 0.300 *	−0.55 *
	2	y = 15.78 − 1.54x R^2^ = 0.064	−0.25
	3	y = 12.71 − 1.09x R^2^ = 0.048	−0.22
	4	y = 12.55 + 2.12x R^2^ = 0.047	0.22

The sign * in the rows represents a significant difference at *p* < 0.05.

**Table 2:** Coefficients of correlation between *MyHC*, MRFs, and *MSTN* paralogs, gene expression levels in the white muscles of salmon yearlings in the HL group.

Parameter	*MyoG*	*Myf5*	*MyoD1a*	*MyoD1b*	*MyoD1c*	*MSTN1a*	*MSTN1b*
*MyHC*	0.23	−0.12	0.36 *	−0.23	0.01	0.27	0.51 *
	(*p* = 0.13)	(*p* = 0.43)	(*p* = 0.04)	(*p* = 0.18)	(*p* = 0.97)	(*p* = 0.10)	(*p* = 0.00)
*MyoG*		−0.17	0.64 *	−0.20	−0.24	−0.35 *	0.27
		(*p* = 0.27)	(*p* = 0.00)	(*p* = 0.22)	(*p* = 0.11)	(*p* = 0.03)	(*p* = 0.10)
*Myf5*			−0.19	0.47 *	0.51 *	0.26	−0.13
			(*p* = 0.27)	(*p* = 0.00)	(*p* = 0.00)	(*p* = 0.10)	(*p* = 0.43)
*MyoD1a*				0.20	−0.17	−0.39 *	0.25
				(*p* = 0.28)	(*p* = 0.34)	(*p* = 0.03)	(*p* = 0.18)
*MyoD1b*					0.66 *	0.01	−0.19
					(*p* = 0.00)	(*p* = 0.95)	(*p* = 0.28)
*MyoD1c*						0.41 *	−0.04
						(*p* = 0.01)	(*p* = 0.80)
*MSTN1a*							0.46 *
							(*p* = 0.01)

The sign * in the rows represents a significant difference at *p* < 0.05.

**Table 3:** Coefficients of correlation between *MyHC*, MRFs, and *MSTN* paralogs, gene expression levels in the white muscles of salmon yearlings in the Group 2 (LD 24:0 May–October 2018).

Parameter	*MyoG*	*Myf5*	*MyoD1a*	*MyoD1b*	*MyoD1c*	*MSTN1a*	*MSTN1b*
*MyHC*	0.33 *	−0.39 *	0.20	−0.37 *	−0.02	0.35 *	0.35 *
	(*p* = 0.04)	(*p* = 0.01)	(*p* = 0.25)	(*p* = 0.03)	(*p* = 0.90)	(*p* = 0.03)	(*p* = 0.04)
*MyoG*		−0.12	0.69 *	0.18	−0.21	−0.07	0.29
		(*p* = 0.45)	(*p* = 0.00)	(*p* = 0.29)	(*p* = 0.17)	(*p* = 0.68)	(*p* = 0.08)
*Myf5*			0.26	0.45 *	0.30 *	−0.03	−0.16
			(*p* = 0.12)	(*p* = 0.00)	(*p* = 0.04)	(*p* = 0.84)	(*p* = 0.35)
*MyoD1a*				0.14	0.22	−0.15	0.10
				(*p* = 0.44)	(*p* = 0.19)	(*p* = 0.39)	(*p* = 0.56)
*MyoD1b*					0.53 *	0.23	0.40 *
					(*p* = 0.00)	(*p* = 0.19)	(*p* = 0.02)
*MyoD1c*						0.30 *	0.38 *
						(*p* = 0.047)	(*p* = 0.02)
*MSTN1a*							0.56 *
							(*p* = 0.00)

The sign * in the rows represents a significant difference at *p* < 0.05.

**Table 4:** Coefficients of correlation between *MyHC*, MRFs, and *MSTN* paralogs, gene expression levels in the white muscles of salmon yearlings in the Group 3 (LD 24:0 August–November 2017, May–October 2018).

Parameter	*MyoG*	*Myf5*	*MyoD1a*	*MyoD1b*	*MyoD1c*	*MSTN1a*	*MSTN1b*
*MyHC*	0.03	0.14	0.35 *	0.28	0.25	0.40 *	−0.13
	(*p* = 0.87)	(*p* = 0.41)	(*p* = 0.04)	(*p* = 0.14)	(*p* = 0.12)	(*p* = 0.02)	(*p* = 0.46)
*MyoG*		−0.19	0.65 *	0.10	−0.29	−0.23	0.37 *
		(*p* = 0.25)	(*p* = 0.00)	(*p* = 0.59)	(*p* = 0.07)	(*p* = 0.18)	(*p* = 0.03)
*Myf5*			−0.08	0.71 *	0.23	−0.12	−0.02
			(*p* = 0.63)	(*p* = 0.00)	(*p* = 0.12)	(*p* = 0.48)	(*p* = 0.92)
*MyoD1a*				0.06	−0.08	0.03	0.11
				(*p* = 0.77)	(*p* = 0.61)	(*p* = 0.87)	(*p* = 0.54)
*MyoD1b*					0.46 *	0.11	−0.04
					(*p* = 0.01)	(*p* = 0.54)	(*p* = 0.83)
*MyoD1c*						0.35 *	−0.10
						(*p* = 0.02)	(*p* = 0.56)
*MSTN1a*							0.15
							(*p* = 0.42)

The sign * in the rows represents a significant difference at *p* < 0.05.

**Table 5:** Coefficients of correlation between *MyHC*, MRFs, and *MSTN* paralogs, gene expression levels in the white muscles of salmon yearlings in the Group 4 (LD 24:0 August 2017–October 2018).

Parameter	*MyoG*	*Myf5*	*MyoD1a*	*MyoD1b*	*MyoD1c*	*MSTN1a*	*MSTN1b*
*MyHC*	0.32 *	0.19	0.32	0.17	0.27	0.45 *	0.63 *
	(*p* = 0.04)	(*p* = 0.26)	(*p* = 0.06)	(*p* = 0.33)	(*p* = 0.10)	(*p* = 0.00)	(*p* = 0.00)
*MyoG*		0.18	0.56 *	−0.15	−0.13	−0.08	0.53 *
		(*p* = 0.26)	(*p* = 0.00)	(*p* = 0.39)	(*p* = 0.40)	(*p* = 0.61)	(*p* = 0.00)
*Myf5*			0.41 *	0.58 *	0.17	0.06	0.40 *
			(*p* = 0.02)	(*p* = 0.00)	(*p* = 0.30)	(*p* = 0.71)	(*p* = 0.02)
*MyoD1a*				0.59 *	−0.36 *	−0.17	0.62 *
				(*p* = 0.00)	(*p* = 0.04)	(*p* = 0.36)	(*p* = 0.00)
*MyoD1b*					0.04	0.17	0.58 *
					(*p* = 0.80)	(*p* = 0.32)	(*p* = 0.00)
*MyoD1c*						0.29	0.08
						(*p* = 0.08)	(*p* = 0.67)
*MSTN1a*							0.21
							(*p* = 0.23)

The sign * in the rows represents a significant difference at *p* < 0.05.

**Table 6:** The weight and length measurements of salmon taken for RT-qPCR analysis.

Date	Group	Regime	n	Average Weight, g	Average Length, cm
12 May	1	HL	10	4.20 ± 0.16	7.75 ± 0.08
	2	LD 24:0 (May–October 2018)	10	4.22 ± 0.16	7.66 ± 0.11
	3	LD 24:0 (August–November 2017 and May–October 2018)	10	4.23 ± 0.19	7.76 ± 0.13
	4	LD 24:0 (August 2017–October 2018)	10	4.22 ± 0.17	7.64 ± 0.10
13 June	1	HL	10	7.55 ± 0.45	8.93 ± 0.13
	2	LD 24:0 (May–October 2018)	10	8.15 ± 0.39	9.13 ± 0.15
	3	LD 24:0 (August–November 2017 and May–October 2018)	10	8.17 ± 0.40	9.17 ± 0.20
	4	LD 24:0 (August 2017–October 2018)	10	8.39 ± 0.33	9.19 ± 0.14
16 July	1	HL	10	14.15 ± 0.74	10.53 ± 0.17
	2	LD 24:0 (May–October 2018)	10	17.06 ± 0.85	11.21± 0.18
	3	LD 24:0 (August–November 2017 and May–October 2018)	10	17.39 ± 1.03	11.16 ± 0.23
	4	LD 24:0 (August 2017–October 2018)	10	16.85 ± 0.77	11.09 ± 0.16
15 August	1	HL	10	20.10 ± 1.23	11.94 ± 0.27
	2	LD 24:0 (May–October 2018)	10	22.94 ± 0.93	12.33 ± 0.14
	3	LD 24:0 (August–November 2017 and May–October 2018)	10	23.00 ± 1.20	12.24 ± 0.23
	4	LD 24:0 (August 2017–October 2018)	10	21.86 ± 2.44	12.13 ± 0.42
14 September	1	HL	10	30.31 ± 2.34	13.91 ± 0.37
	2	LD 24:0 (May–October 2018)	10	31.18 ± 1.43	13.89 ± 0.25
	3	LD 24:0 (August–November 2017 and May–October 2018)	10	32.80 ± 2.28	14.11 ± 0.35
	4	LD 24:0 (August 2017–October 2018)	10	32.55 ± 2.44	14.08 ± 0.33
15 October	1	HL	10	33.58 ± 2.56	15.85 ± 0.29
	2	LD 24:0 (May–October 2018)	10	31.90 ± 2.82	14.13 ± 0.41
	3	LD 24:0 (August–November 2017 and May–October 2018)	10	33.24 ± 2.58	14.21 ± 0.40
	4	LD 24:0 (August 2017–October 2018)	10	34.74 ± 2.19	14.36 ± 0.29

Values are means ± SE.

**Table 7:** Oligonucleotide primers used for RT-qPCR amplification.

Gene	Primer Sequence (5′–3′)	Size of Amplified Fragment, bp	GenBank Accession No.
*Ef-1α*	F: TTGCTGGTGGTGTTGGTGAG R: AAACGCTTCTGGCTGTAGGG	154	AF321836.1
*MyoG*	F: GTGGAGATCCTGAGGAGTGC R: CTCACTCGACGACGAGACC	147	DQ452070
*MyoD1a*	F: TGGACTGCCTATCAAACATCC R: TCTCACTCGCTATGGAACC	123	AJ557148
*MyoD1b*	F: ATTTCGTTCCCTGTCACCTCTG R: ATGTGTTCGTCTTCGTTGTAATGG	152	AJ557150
*MyoD1c*	F: ACGGCGAAAACTACTACCCTTC R: TAGCTGCTTCGTCTTGCGGA	133	DQ366709.1
*Myf5*	F: ACGCCATCCAGTACATCGAG R: AGTCAACCATGCTGTCGGAG	132	DQ452070
*MyHC*	F: TCTCATCCATAGACGCCATC R: AGTTGACTGCCAAGAAGAGG	159	DN164736
*MSTN1a*	F: GATTACACGCCATCAAGTCC R: CTCCATCCTTATTGTCATCTCC	159	AJ344158
*MSTN1b*	F: TCTGAGTTTTATGGTTGCTTTCGG R: TTGTGACTTGATGGCGTGTAATC	151	NM_001123634.1

Forward (F) and reverse (R) primer sequences (5′–3′), bp—base pairs; *Ef-1a*—elongation factor 1α; *Myf5*—myogenic factor 5; *MyoD1*—myoblast determination protein 1; *MyoG*—myogenin; *MyHC*—myosin heavy chain; *MSTN*—myostatin.

## References

[1] Johnston I.A. (2006). Environment and plasticity of myogenesis in teleost fish. J. Exp. Biol..

[2] Watabe S., Johnston I.A. (2001). Myogenic regulatory factors. Fish Physiology-Muscle Development and Growth.

[3] Johansen K.A., Overturf K. (2005). Quantitative expression analysis of genes affecting muscle growth during development of rainbow trout (*Oncorhynchus mykiss*). Mar. Biotech..

[4] Watabe S., Ikeda D. (2006). Diversity of the pufferfish Takifugu rubripes fast skeletal myosin heavy chain genes. Comp. Biochem. Physiol. Part. D Gen. Prot..

[5] Hevrøy E.M., Jordal A.O., Hordvik I., Espe M., Hemrea G.-I., Olsvik P.A. (2006). Myosin heavy chain mRNA expression correlates higher with muscle protein accretion than growth in Atlantic salmon *Salmo salar*. Aquaculture.

[6] Dhillon R.S., Esbaugh Y.S., Wang B.L. (2009). Characterization and expression of a myosin heavy–chain isoform in juvenile walleye *Sander vitreus*. J. Fish. Biol..

[7] Gabillard J.C., Biga P.R., Rescan P.Y., Seiliez I. (2013). Revisiting the paradigm of myostatin in vertebrates: Insights from fishes. Gen. Comp. Endocrinol..

[8] McCroskery S., Thomas M., Maxwell L., Sharma M., Kambadur R. (2003). Myostatin negatively regulates satellite cell activation and self-renewal. J. Cell Biol..

[9] Ostaszewska T., Dabrowski K., Wegner A., Krawiec M. (2008). The effects of feeding on muscle growth dynamics and the proliferation of myogenic progenitor cells during pike perch development (*Sander lucioperca*). J. World Aquac. Soc..

[10] Chapalamadugu K.C., Robison B.D., Drew R.E., Powell M.S., Hill R.A., Amberg J.J., Rodnick K.J., Hardy R.W., Hill M.L., Murdoch G.K. (2009). Dietary carbohydrate level affects transcription factor expression that regulates skeletal muscle myogenesis in rainbow trout. Comp. Biochem. Physiol. B.

[11] Hagen Ø., Fernandes J.M.O., Solberg C., Johnston I.A. (2009). Expression of growth-related genes in muscle during fasting and refeeding of juvenile Atlantic halibut, *Hippoglossus hippoglossus* L.. Comp. Biochem. Physiol. B.

[12] Johnston I.A., McLay H.A., Abercromby M., Robins D. (2000). Early thermal experience has different effects on growth and muscle fibre recruitment in spring- and autumn-running Atlantic salmon populations. J. Exp. Biol..

[13] Wilkes D., Xie S.Q., Stickland N.C., Alami-Durante H., Goldspink G. (2001). Temperature and myogenic factor transcript levels during early development determines muscle growth potential in rainbow trout (*Oncorhynchus mykiss*) and sea bass (*Dicentrarchus labrax*). J. Exp. Biol..

[14] López-Albors O., Abdel I., Periago M.J., Ayala M.D., Alcazar A.G., Graciá C.M., Nathanailides C., Vázquez J.M. (2008). Temperature influence on the white muscle growth dynamics of the sea bass *Dicentrarchus labrax*, L. Flesh quality implications at commercial size. Aquaculture.

[15] Johnston I.A., Manthri S., Smart A., Campbell P., Nickell D., Alderson R. (2003). Plasticity of muscle fibre number in seawater stages of Atlantic salmon in response to photoperiod manipulation. J. Exp. Biol..

[16] Nagasawa K., Giannetto A., Fernandes J.M.O. (2012). Photoperiod influences growth and mll (mixed-lineage leukaemia) expression in Atlantic cod. PLoS ONE.

[17] Ayala M.D., Abellan E., Arizcun M., Garcia-Alcazar A., Navarro F., Blanco A., Lopez-Albors O.M. (2013). Muscle development and body growth in larvae and early post-larvae of shi drum, *Umbrina cirrosa* L., reared under different larval photoperiod: Muscle structural and ultrastructural study. Fish. Physiol. Biochem..

[18] Boeuf G., Falcon J. (2002). Photoperiod and growth in fish. Vie Milieu.

[19] Taylor J.F., North B.P., Porter M.J.R., Bromage N.R., Migaud H. (2006). Photoperiod can be used to enhance growth and improve feeding efficiency in farmed rainbow trout, *Oncorhynchus mykiss*. Aquaculture.

[20] Taylor J., Migaud H. (2009). Timing and duration of constant light affects rainbow trout (*Oncorhynchus mykiss*) growth during autumn–spring grow-out in freshwater. Aquac. Res..

[21] Noori A., Mojazi Amiri B., Mirvaghefi A., Rafiee G., KalvaniNeitali B. (2015). Enhanced growth and retarded gonadal development of farmed rainbow trout, *Oncorhynchus mykiss* (Walbaum) following a long-day photoperiod. Aquac. Res..

[22] Imsland A.K.D., Roth B., Fjelldal P.G., Stefansson S.O., Handeland S., Mikalsen B. (2017). The effect of continuous light at low temperatures on growth in Atlantic salmon reared in commercial size sea pens. Aquaculture.

[23] Saunders R.L., Henderson E.B., Harmon P.R. (1985). Effects of photoperiod on juvenile growth and smolting of Atlantic salmon and subsequent survival and growth in sea cages. Aquaculture.

[24] Saunders R.L., Henderson E.B. (1988). Effects of constant day length on sexual maturation and growth of Atlantic salmon (*Salmo salar*) parr. Can. J. Fish. Aquatic Sci..

[25] Handeland S.O., Björnsson B.T., Arnesen A.M., Stefansson S.O. (2003). Seawater adaptation and growth of post-smolt Atlantic salmon (*Salmo salar* L.) of wild and farmed strain. Aquaculture.

[26] Imsland A.K., Folkvord A., Stefansson S.O. (1995). Growth, oxygen consumption and activity of juvenile turbot (*Scopthalmus maximus* L.) reared under different temperatures and photoperiods. Neth. J. Sea Res..

[27] Petit G., Beauchaud M., Attia J., Buisson B. (2003). Food intake and growth of largemouth bass (*Micropterus salmoides*) held under alternated light/dark cycle (12L:12D) or exposed to continuous light. Aquaculture.

[28] Jonassen T.M., Imsland A.K., Kadowaki S., Stefabsson S.O. (2000). Interaction of temperature and photoperiod on growth of Atlantic halibut *Hippoglossus hippoglossus* L.. Aquac. Res..

[29] Kissil G.W., Lupatsch I., Elizur A., Zohar Y. (2001). Long photoperiod delayed spawning and increased somatic growth in gilthead sea bream (*Sparus aurata*). Aquaculture.

[30] Nordgarden U., Oppedal F., Taranger G.L., Hemre G.I., Hansen T. (2003). Seasonally changing metabolism in Atlantic salmon (*Salmo salar* L.) I–Growth and feed conversion ratio. Aquac. Nutr..

[31] Björnsson B.T., Hemre G.-I., Bjørnevik M., Hansen T. (2000). Photoperiod regulation of plasma growth hormone levels during induced smoltification of underyearling Atlantic salmon. Gen. Comp. Endocrinol..

[32] McCormick S.D., Moriyama S., Björnsson B.T. (2000). Low temperature limits photoperiod control of smolting in Atlantic salmon through endocrine mechanisms. Am. J. Physiol. Regul. Integr. Comp. Physiol..

[33] Reinecke M., Björnsson B.T., Dickhoff W.W., McCormick S.D., Navarro I., Power D.M., Gutiérrez J. (2005). Growth hormone and insulin-like growth factors in fish: Where we are and where to go. Gen. Comp. Endocrinol..

[34] Kwasek K., Wick M., Dabrowski K., Kestemont P., Dabrowski K., Summerfelt R.C. (2015). Muscle protein characteristic and its association with faster growth in percids and other teleosts. Biology and Culture of Percid Fishes, eBook.

[35] Hansen T., Stefansson S., Taranger G.L. (1992). Growth and sexual maturation in Atlantic salmon, *Salmon salar* L., reared in sea cages at two different light regimes. Aquac. Res..

[36] Bromage N., Randall C., Davies B., Thrush M., Duston J., Carillo M., Zanuy S. (1993). Photoperiodism and the control of reproduction and development in farmed fish. Aquac. Fundam. Appl. Res..

[37] Nemova N.N., Nefedova Z.A., Pekkoeva S.N., Voronin V.P., Shulgina N.S., Churova M.V., Murzina S.A. (2020). The Effect of the Photoperiod on the Fatty Acid Profile and Weight in Hatchery-Reared Underyearlings and Yearlings of Atlantic Salmon *Salmo salar* L.. Biomolecules.

[38] Churova M.V., Meshcheryakova O.V., Veselov A.E., Nemova N.N. (2015). Activity of enzymes involved in the energy and carbohydrate metabolism and the level of some molecular-genetic characteristics in young salmons (*Salmo salar* L.) with different age and weight. Russ. J. Dev. Biol..

[39] Johnston I.A., Manthri S., Bickerdike R., Dingwall A., Luijkx R., Campbell P., Nickell D., Alderson R. (2004). Growth performance, muscle structure and flesh quality in out-of-season Atlantic salmon (*Salmo salar*) smolts reared under two different photoperiod regimes. Aquaculture.

[40] Macqueen D.J., Johnston I.A. (2006). A novel salmonid myoD gene is distinctly regulated during development and probably arose by duplication after the genome tetraploidization. FEBS Lett..

[41] Bower N.I., Johnston I.A. (2010). Paralogs of Atlantic salmon myoblast determination factor genes are distinctly regulated in proliferating and differentiating myogenic cells. Am. J. Phys. Regul. Integr. Comp. Phys..

[42] de Almeida F.L.A., Carvalho R.F., Pinhal D., Padovani C.R., Martins C., Dal Pai-Silva M. (2008). Differential expression of myogenic regulatory factor MyoD in pacu skeletal muscle (*Piaractus mesopotamicus* Holmberg 1887: Serrasalminae, Characidae, Teleostei) during juvenile and adult growth phases. Micron.

[43] Takata R., Nakayama C.L., e Silva W.D.S., Bazzoli N., Luz R.K. (2018). The effect of water temperature on muscle cellularity and gill tissue of larval and juvenile Lophiosilurus alexandri, a Neotropical freshwater fish. J. Therm. Biol..

[44] Valente L.M., Bower N.I., Johnston I.A. (2012). Postprandial expression of growth-related genes in Atlantic salmon (*Salmo salar* L.) juveniles fasted for 1 week and fed a single meal to satiation. Br. J. Nutr..

[45] Danzmann R.G., Kocmarek A.L., Norman J.D., Rexroad C.E., Palti Y. (2016). Transcriptome profiling in fast versus slow-growing rainbow trout across seasonal gradients. BMC Genom..

[46] Rowlerson A., Veggetti A., Johnston I.A. (2001). Cellular mechanisms of post-embryonic muscle growth in aquaculture species. Fish Physiol.: Muscle Development and Growth.

[47] Ayala M.D., Lopez-Albors O., Gil F., Garcıa-Alcazar A., Abellán E., Alarcon J.A., Álvarez M.C., Ramírez-Zarzosa G., Moreno F. (2001). Temperature effects on muscle growth in two populations (Atlantic and Mediterranean) of sea bass, *Dicentrarchus labrax* L.. Aquaculture.

[48] Berkes C.A., Tapscott S.J. (2005). MyoD and the transcriptional control of myogenesis. Semin. Cell Dev. Biol..

[49] Churova M.V., Meshcheryakova O.V., Veselov A.E., Efremov D.A., Nemova N.N. (2017). Activity of metabolic enzymes and muscle-specific gene expression in parr and smolts Atlantic salmon *Salmo salar* L. of different age groups. Fish Physiol. Biochem..

[50] De Almeida F.L.A., Pessotti N.S., Pinhal D., Padovani C.R., de Jesus Leitão N., Carvalho R.F., Martins C., Portella M.C., Pai-Silva M.D. (2010). Quantitative expression of myogenic regulatory factors MyoD and myogenin in pacu (*Piaractus mesopotamicus*) skeletal muscle during growth. Micron.

[51] Churova M.V., Shulgina N.S., Kuritsyn A., Krupnova M.Y., Nemova N.N. (2020). Muscle-specific gene expression and metabolic enzyme activities in Atlantic salmon Salmo salar L. fry reared under different photoperiod regimes. Comp. Biochem. Physiol. B Biochem. Mol. Biol..

[52] Johnston I.A., Johnston I.A. (2001). Genetic and environmental determinants of muscle growth patterns. Fish Physiology.

[53] Weber T.E., Bosworth B.G. (2005). Effects of 28 day exposure to cold temperature or feed restriction on growth, body composition, and expression of genes related to muscle growth and metabolism in channel catfish. Aquaculture.

[54] Garikipati D.K., Rodgers B.D. (2012). Myostatin inhibits myosatellite cell proliferation and consequently activates differentiation: Evidence for endocrine-regulated transcript processing. J. Endocrinol..

[55] Seiliez I., Sabin N., Gabillard J.C. (2012). Myostatin inhibits proliferation but not differentiation of trout myoblasts. Mol. Cell. Endocrinol..

[56] Joulia D., Bernardi H., Garandel V., Rabenoelina F., Vernus B., Cabello G. (2003). Mechanisms involved in the inhibition of myoblast proliferation and differentiation by myostatin. Exp. Cell Res..

[57] Johnston I.A., Macqueen D.J., Watabe S., Tsukamoto K., Kawamura T., Takeuchi T., Beard T.D., Kaiser M.J. (2008). Molecular biotechnology of development and growth in fish muscle. Fisheries for Global Welfare and Environment: Memorial Book of the 5th World Fisheries Congress.

[58] Johansen K.A., Overturf K. (2006). Alterations in expression of genes associated with muscle metabolism and growth during nutritional restriction and refeeding in rainbow trout. Comp. Biochem. Physiol. B Biochem. Mol. Biol..

[59] Livak K.J., Schmittgen T.D. (2001). Analysis of relative gene expression data using real-time quantitative PCR and the 2−ΔΔCt method. Methods.

